# Identification of an IGF1 mutation (c.15+3G>A) in familial osteonecrosis of the femoral head: A case report

**DOI:** 10.1097/MD.0000000000023705

**Published:** 2021-01-08

**Authors:** Qi Xu, Da Song, Zhensong Wu, Bo Fu, Juan Zheng, Rongrong Li, Han Yin, Shuangfeng Chen, Dawei Wang

**Affiliations:** a Department of Joint Surgery, Liaocheng People's Hospital, Liaocheng; b Department of Joint Surgery, Zaozhuang Municipal Hospital, Zaozhuang; c Department of Central Laboratory; d Department of Joint Laboratory for Translational Medicine Research, Liaocheng People's Hospital, Liaocheng, China.

**Keywords:** detection, IGF1 mutation, osteonecrosis of the femoral head, treatment

## Abstract

**Rationale:**

Osteonecrosis of the femoral head is a common orthopedic disease. Some studies have shown that osteonecrosis of the femoral head is related to susceptibility genes, but there are few reports of familial osteonecrosis of the femoral head. Here, we reported two first-degree relatives with osteonecrosis of the femoral head.

**Patient concerns:**

A 27-year-old man with bilateral hip pain was diagnosed with osteonecrosis of the femoral head. The patient's mother also had a history of this disease.

**Diagnoses:**

Whole exome sequencing revealed the same mutation (c.15+3G>A) in the insulin-like growth factor 1 (IGF1) gene of the proband and his mother but not in his elder sister.

**Interventions:**

The patient underwent bilateral total hip arthroplast.

**Outcomes:**

The patient recovered well, and was discharged.

**Lessons:**

We found a heterozygous mutation (c.15+3G>A) in IGF1 in this family, which could be related to osteonecrosis of the femoral head. Early genetic counseling and gene locus detection could, thus, prove helpful for early diagnosis of osteonecrosis of the femoral head.

## 1 Introduction

Osteonecrosis of the femoral head is a refractory disease caused by various factors that lead to vascular blockage in specific areas, resulting in insufficient blood supply, limited transport of oxygen and nutrients, and the eventual death of bone cells, deformation, and collapse of the femoral head, and dyskinesia of the hip joint.^[1]^ The development of this disease causes irreversible damage to the femoral head, limiting the patient's lifestyle, and severely affecting the patient's quality of life. The incidence of osteonecrosis of the femoral head is increasing each year in China. Approximately 200,000 new patients are diagnosed every year, accounting for one-fifth of all patients with femoral head necrosis worldwide.^[2]^ Although some studies showed that the pathogenesis of osteonecrosis of the femoral head is related to trauma, corticosteroid use, alcohol abuse, and other factors, the specific pathogenesis is unclear.^[3]^ In recent years, some studies showed that genetic factors promote the occurrence of osteonecrosis of the femoral head.^[4]^

There are few reports of familial osteonecrosis of the femoral head, with most focusing on the gene locus of COL2A1.^[5]^ Here, we report two first-degree relatives who were diagnosed with osteonecrosis of the femoral head and underwent surgical resection. Genes related to osteonecrosis of the femoral head were analyzed by full whole exome sequencing (WES), which revealed insulin-like growth factor 1 (IGF1) as a potential marker for genetic counseling and molecular diagnosis of osteonecrosis of the femoral head.

## 2 Case report

### 2.1 Ethics statement

This study was approved by the Ethics Committee of our hospital (ethical approval number: 2018010). Written informed consent was obtained from the patient for publication of this case report and accompanying images.

### 2.2 Patient features and investigations

**Figure 1:**
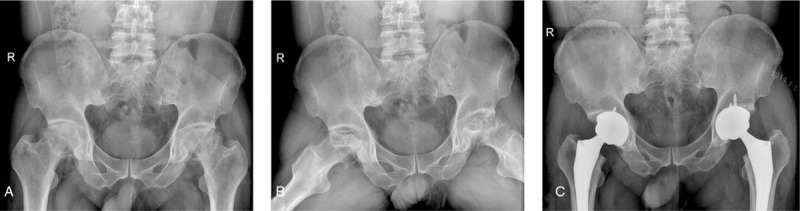
X-ray examination of pelvis (A and B) before and (C) after operation.

A 28-year-old young man visited Hospital in January 2018 with a complaint of bilateral hip pain. The patient had experienced hip pain a year prior, which was aggravated after exercise, and relieved after rest, without lower limb numbness and radiation pain. He had no history of other diseases and no trauma, corticosteroid use, alcohol abuse, or other causes. Physical examination showed tenderness at the midpoint of the bilateral groin, obvious limitation of bilateral hip joint movement, Patrick test (+), and no obvious abnormality of the other limbs. Laboratory tests revealed slightly increased levels of interleukin-6, whereas other indices were normal. Pelvic X-ray examination showed bilateral collapse and fragmentation of the femoral head. Hyperosteogeny at the edge of the femoral head and acetabulum, unsmooth appearance, and uneven subchondral bone mineral density were observed (Fig. 1). Based on the uneven narrowing of the joint space, the patient was diagnosed with stage IV osteonecrosis of the femoral head.

### 2.3 Treatment

**Figure 2:**
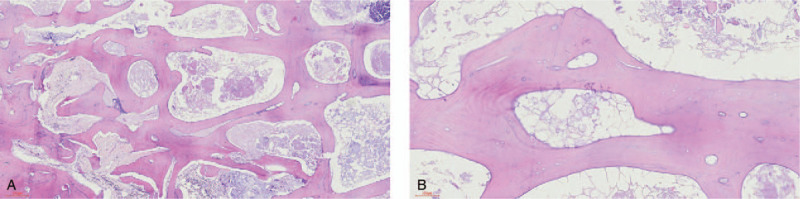
Histopathological analysis of femoral head by hematoxylin-eosin staining. Microscopic examination revealed thinner bone trabeculae, different shapes, fat necrosis in the local medullary cavity, calcium deposition, and wrapped in necrotic bone.

After the patient's consent and preoperative examination, we performed bilateral total hip arthroplasty. After operation, the necrotic hip joint was divided into two parts with a bone knife. Dead bone tissue was collected and sent to the pathology department for hematoxylin-eosin staining (Fig. 2). Microscopic examination revealed thinner bone trabeculae, different shapes, fat necrosis in the local medullary cavity, calcium deposition, and wrapped in necrotic bone. These pathological characteristics support the diagnosis of osteonecrosis of the femoral head.

### 2.4 Outcome and follow-up

After surgery, the patient was transferred to the joint surgery ward. The patient recovered well and could walk after 2 days. No obvious abnormality was found in any laboratory examinations or on pelvic X-ray films (Fig. 1).

### 2.5 Family history

**Figure 3:**
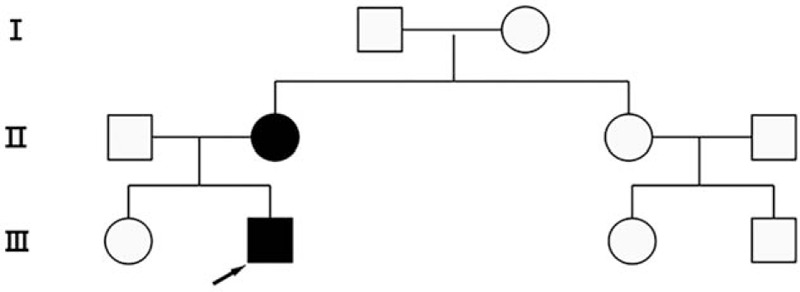
Pedigree of the family with osteonecrosis of the femoral head. Arrow indicates the proband.

A family pedigree across 3 generations was constructed for the patient (Fig. 3). The mother (55 years old) of the proband also suffered from osteonecrosis of the femoral head and total hip arthroplasty was performed 7 years ago. However, the elder sister (30 years old) of the proband showed no sign of osteonecrosis of the femoral head in imaging examination.

### 2.6 WES

**Figure 4:**
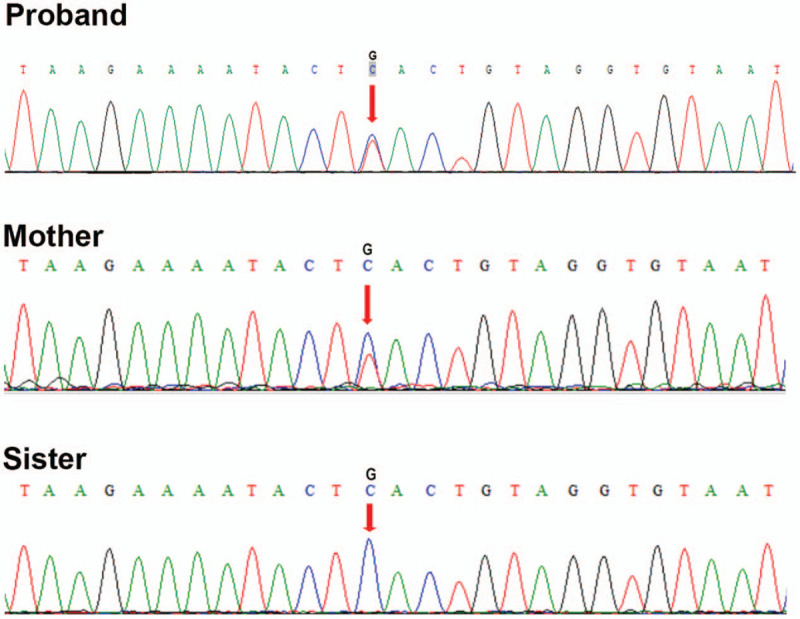
IGF1 mutations in the patients and his family. Both osteonecrosis of the femoral head cases in the family (the proband and her mother) carried the same IGF1 intron 1 heterozygous mutation (c.15+3G>A), and his sister did not carry the mutation.

Peripheral blood samples were collected from the patient, his mother, and his sister. Specimens could not be obtained from the patient's father and grandparents. We extracted the DNA from the three blood samples and performed WES to identify the possible pathogenic variants. Bioinformatic analyses of WES revealed a heterozygous mutation (c.15+3G>A) within intron 1 in the IGF1 gene in both the patient and his mother, but not in his sister. These results were confirmed by Sanger sequencing (Fig. 4).

### 2.7 Quantitative real-time PCR (qPCR)analysis of IGF1 gene transcripts

**Figure 5:**
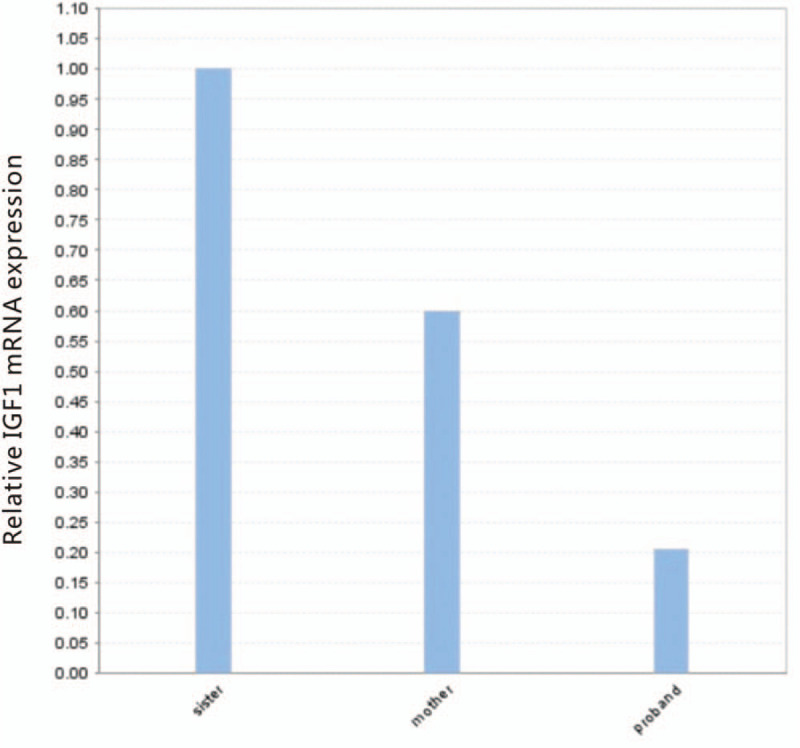
Quantitative real-time PCR (qPCR)analysis of IGF1 gene transcripts.

Total RNA was extracted from peripheral blood of probands, mothers and sisters, and cDNA was synthesized by reverse transcription followed by real-time PCR experiments to detect the differences in the expression of amplified IGF1 gene products in mRNA in different groups. The results showed that the relative expression of mRNA was significantly lower in the proband and mother compared with the sister. Therefore, we speculated that this mutation resulted in abnormal cleavage of the mRNA (Fig. 5).

## 3 Discussion

Osteonecrosis of the femoral head is a common disease in the field of orthopedics, typically occurring at ages between 35 and 50 years old. Factors inducing osteonecrosis of the femoral head are numerous and not always obvious, but the condition is very serious. This disease also shows genetic susceptibility. In this study, we found a new IGF1 mutation in a Chinese family by WES. We verified this mutation by Sanger sequencing, which supported that the IGF1 mutation (c.15+3G>A) was the pathogenic gene in this family.

IGF1 is a growth factor with important biological functions, such as promoting the growth and development of postnatal bones. It acts on osteocytes through autocrine and paracrine signaling, promotes the proliferation of osteoblasts and osteoclasts, regulates the resorption of bone, and participates in bone remodeling.^[6]^ Many studies have shown that the loss of IGF1 affects bone development and leads to necrosis of bone cells. The cytoskeleton of IGF1 knockout mice was smaller than that of ordinary mice, their osteogenic ability was significantly weakened, and the osteocytes were more prone to apoptosis.^[7]^ IGF1 can reverse osteocyte apoptosis through the PI3K/AKT pathway.^[8]^ Intracellular calcium Ca2+ signaling plays an important role in the formation, activation, and apoptosis of osteocytes, and IGF1 binds to Ca2+ receptors to open calcium channels.^[9,10]^ In rabbits with steroid-induced osteonecrosis of the femoral head, IGF1 was measured by enzyme-linked immunosorbent assay at 4, 8, and 16 weeks, and osteonecrosis of the femoral head was diagnosed based on magnetic resonance imaging and histological findings. We found that IGF1 began to increase 4 weeks earlier than the abnormal bone marrow tissue in the rabbits.^[11]^ Therefore, IGF1 is closely related to osteonecrosis of the femoral head.

We identified a mutation at the third base in the beginning of intron 1 of IGF1. The mutation occurs at the intron donor site to change G to A, resulting in incorrect site recognition and abnormal mRNA splicing of the gene. This leads to an abnormal quantity or structure of amino acids. Through Hardy–Weinberg equilibrium test and haplotype analysis, Wang et al found that IGF1 polymorphism was closely related to the susceptibility to osteonecrosis of the femoral head.^[12]^ Familial osteonecrosis of the femoral head is typically inherited as an autosomal dominant trait,^[13]^ and thus offspring are likely to have the same genetic mutation leading to the disease. Existing gene detection techniques can be used to provide molecular prenatal diagnosis along with further genetic counseling to enable early and effective interventions and treatments.

We present a familial case of 2 first-degree relatives with osteonecrosis of the femoral head. The proband was diagnosed with the disease, and a gene mutation (c.15+3G>A) was found in IGF1 by WES. His mother showed the same gene mutation, whereas his sister did not. IGF1 mutation may be the disease-causing gene in this family. Therefore, by performing gene analysis in the early stage, susceptibility genes related to osteonecrosis of the femoral head can be screened quickly and accurately. Our study provides a new biological marker for diagnosing osteonecrosis of the femoral head, and a potential new drug intervention target for early treatment of this disease.

## Author contributions

**Conceptualization:** Qi Xu, Dawei Wang.

**Data curation:** Qi Xu, Zhensong Wu, Rongrong Li.

**Formal analysis:** Qi Xu, Juan Zheng.

**Funding acquisition:** Bo Fu.

**Investigation:** Qi Xu.

**Methodology:** Qi Xu, Da Song, Han Yin.

**Project administration:** Qi Xu.

**Resources:** Qi Xu.

**Software:** Qi Xu, Shuangfeng Chen.

**Supervision:** Qi Xu.

**Validation:** Qi Xu.

**Visualization:** Qi Xu.

**Writing – original draft:** Qi Xu, Dawei Wang.

**Writing – review & editing:** Da Song, Zhensong Wu, Dawei Wang.
